# Supplementary material for: Microwave Heating of the Catalyst Bed as a Way of Energy-Saving Oxidative Dehydrogenation of Ethane on a Mo-V-Te-Nb-Ox Catalyst
Source: Nanomaterials (Basel). 2022 Dec 15;12(24):4459. doi: 10.3390/nano12244459 (PMC9787988; doi:10.3390/nano12244459)
Supplement: Supplementary file 1 [file nanomaterials-12-04459-s001.zip › nanomaterials-2087887-supplementary.pdf]

Supplementary Materials

Microwave heating of the catalyst bed as a way of energy-saving oxidative dehydrogenation of ethane on a Mo-V-Te-Nb-Ox catalyst

Alexei Kucherov[1], Nikolai Davshan[1], Elena Finashina[1], Leonid Kustov[1,2,3]

1 Zelinsky Institute of Organic Chemistry, Russian Academy of Sciences, 119991, Moscow, Russian Federation. Fax: +7 499 137 2935; E-mail: akuchero2004@yahoo.com
2 Chemistry Department, Moscow State University, 1 Leninskie Gory, bldg. 3, Moscow, 119992 Russia.
3 National University of Science and Technology MISiS, Leninsky prosp. 4, Moscow, Russia
Correspondence: lmkustov@mail.ru

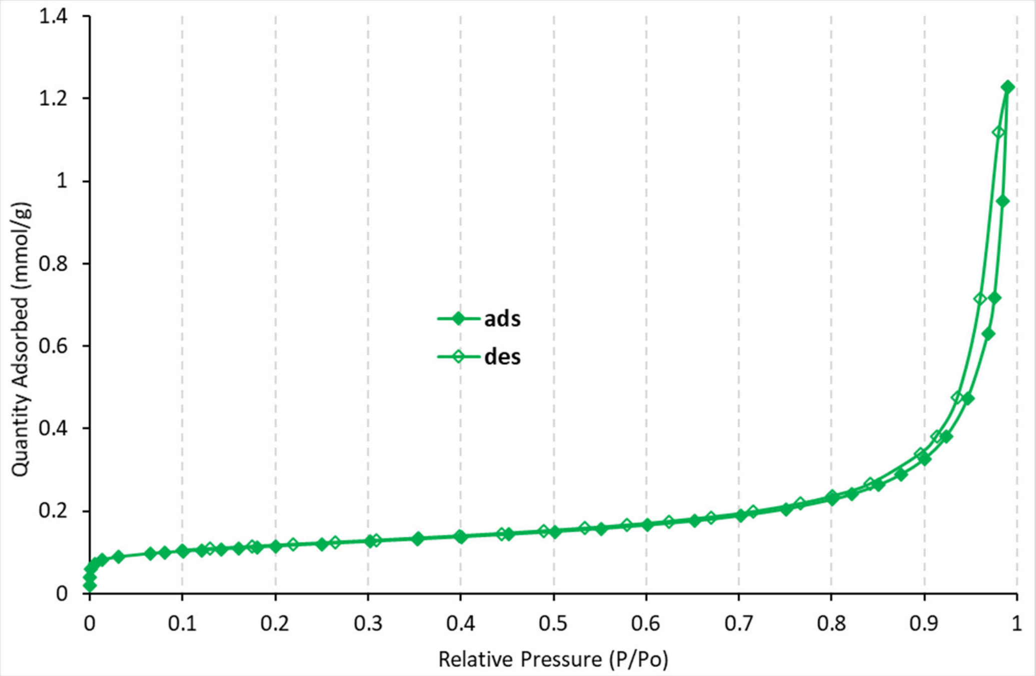

Figure S1. N_2 adsorption isotherms.

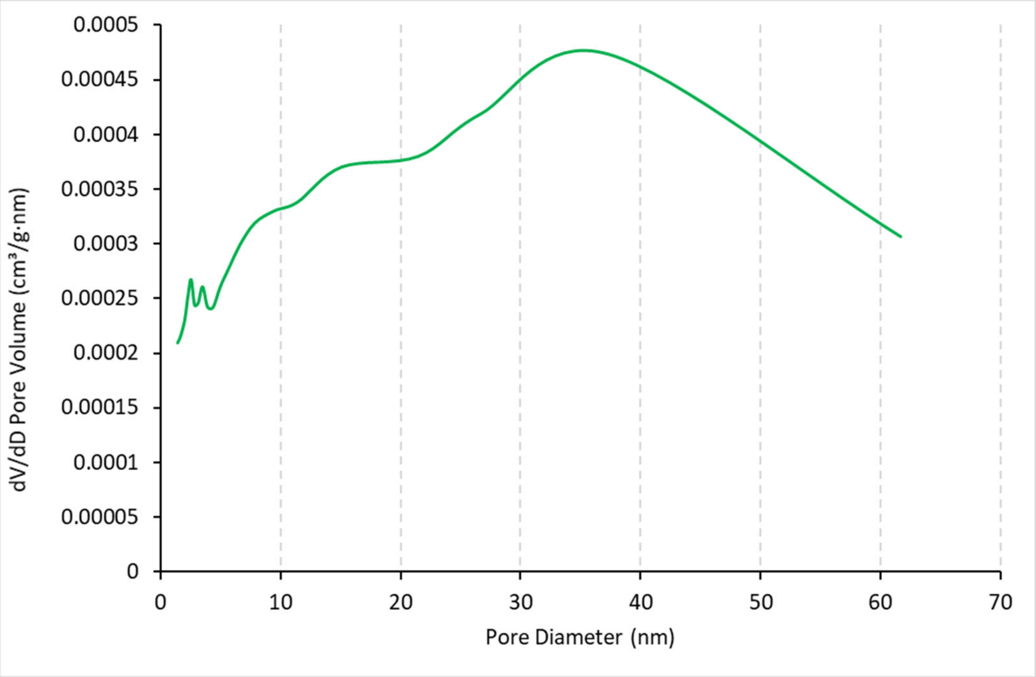

Figure S2. Mesopore size distribution for the Mo-V-Te-Nb-O catalyst (calculation by the BJH method, desorption branch of the isotherm).

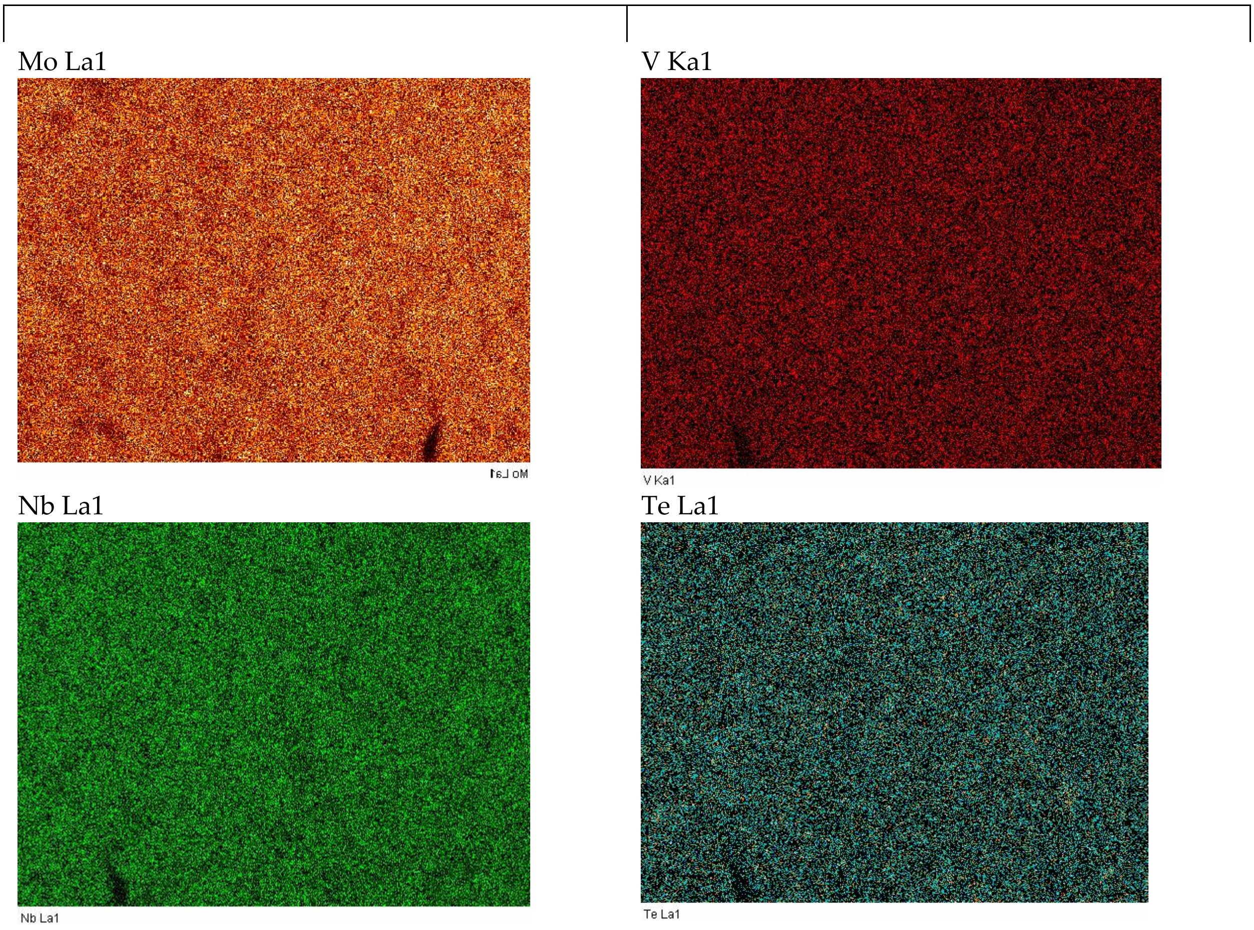

Figure S3. Elements distribution over the crystal phase in the Mo-V-Te-Nb-O catalyst (by EDX).

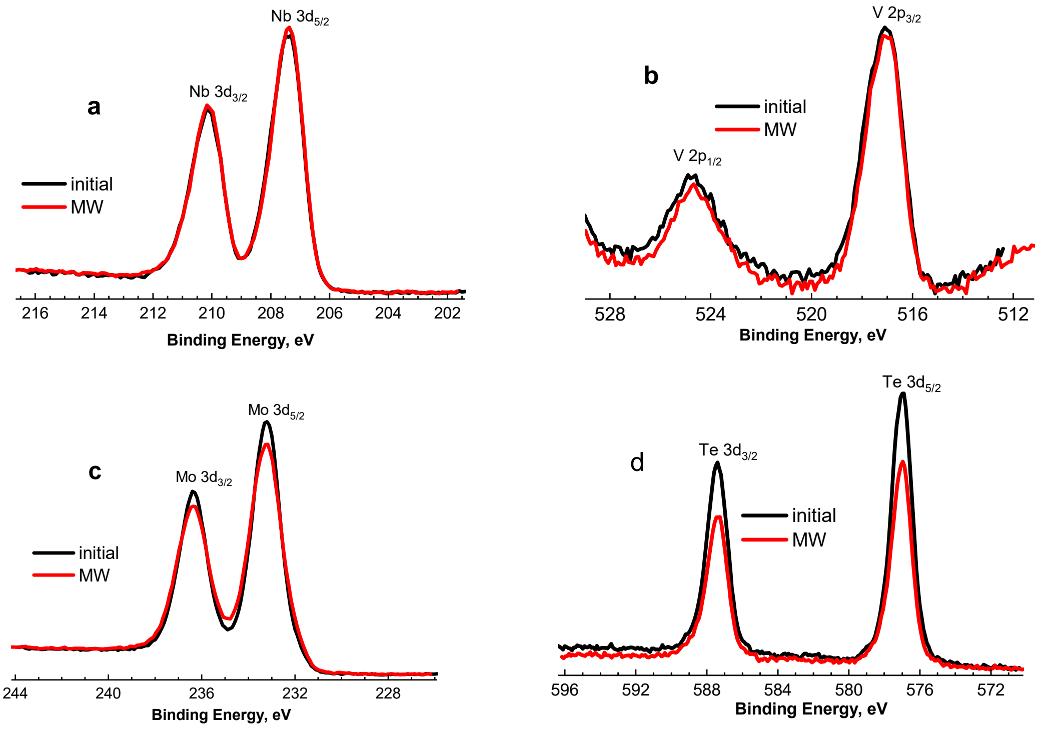

Figure S4. XPS of the MoVTeNbO catalyst: Nb 3d (a), V 2p (b), Mo 3d (c) and Te 3d (d).
